# Preoperative Inflammatory Scores Do Not Accurately Predict Early Recurrence of Pancreatic Ductal Adenocarcinoma After Resection: A Systematic Review and Meta‐Analysis

**DOI:** 10.1002/cam4.70352

**Published:** 2024-10-29

**Authors:** Filipe de Castro e Borges, Elias Khajeh, Rajan Nikbakhsh, Catarina Ribeiro, Markus Maeurer, Carlos Carvalho, Gil Gonçalves, Christoph Berchtold, Markus W. Büchler, Arianeb Mehrabi

**Affiliations:** ^1^ Department of Digestive Surgery, Hepato‐Pancreato‐Biliary Surgery Unit Champalimaud Foundation Lisbon Portugal; ^2^ Faculdade de Medicina Universidade de Lisboa Lisbon Portugal; ^3^ Department of General, Visceral and Transplantation Surgery University Hospital Heidelberg Heidelberg Germany; ^4^ Champalimaud Centre for the Unknown Lisbon Portugal; ^5^ Digestive Unit, Clinical Oncology Champalimaud Clinical Centre Lisbon Portugal

**Keywords:** pancreatic carcinoma, pancreatic surgery, recurrence

## Abstract

**Background:**

Disease recurrence after surgical resection for pancreatic ductal adenocarcinoma can affect more than 50% of patients in the first 12 months after resection. The goal of this current systematic review and meta‐analysis is to assess the ability of preoperative inflammatory scores to predict early recurrence after resection and to identify the best candidates for surgical resection.

**Methods:**

Medline and Web of Science databases were searched for studies reporting inflammatory scores and oncological outcomes in patients with PDAC after curative‐intent resection. The systematic review revealed that the most common scores were modified Glasgow Prognostic Score (mGPS), Prognostic Nutritional Index (PNI), platelet‐to‐lymphocyte ratio (PLR), neutrophil‐to‐lymphocyte ratio (NLR), and Systemic Immune‐Inflammation Index (SII). After, a meta‐analysis was performed to determine the prognostic value of these scores in early recurrence (12 months) after resection. A subgroup analysis was also carried out in patients who had upfront surgery and in those who underwent neoadjuvant chemotherapy. The ROBINS‐I tool was used to assess the risk of bias.

**Results:**

The literature search retrieved 1864 articles, 16 of which were eligible for analysis. The included studies comprised 4460 patients. Nine studies reported outcomes for mGPS, four studies for PNI, seven studies for PLR, eight studies for NLR, and two studies for SII. In the meta‐analysis, mGPS, NLR, and PLR showed significantly higher rates of early recurrence in the high‐score groups compared to the low‐score groups. Analyzing the sensitivity and specificity of these scores showed no significant difference in their diagnostic accuracy (mGPS area under the curve [AUC] = 0.534; NLR AUC = 0.628, and PLR AUC = 0.607). High and low PNI and SII scores demonstrated similar rates of early recurrence.

**Conclusion:**

mGPS, PNI, PLR, NLR, and SII scores did not show a suitable diagnostic accuracy to predict PDAC recurrence in the first 12 months after resection. Therefore, these inflammatory scores should not be used to select the best candidates or to preclude a possible surgical indication.

## Graphical abstract

In this study, we investigated the ability of preoperative inflammatory scores to predict early recurrence after pancreatic resection of pancreatic ductal adenocarcinoma. We found that the modified Glasgow Prognostic Score, Prognostic Nutritional Index, platelet‐to‐lymphocyte ratio, neutrophil‐to‐lymphocyte ratio, and Systemic Immune‐Inflammation Index did not show suitable diagnostic accuracy to predict recurrence in the first 12 months after resection. These inflammatory scores are not helpful in selecting the best surgical candidates.

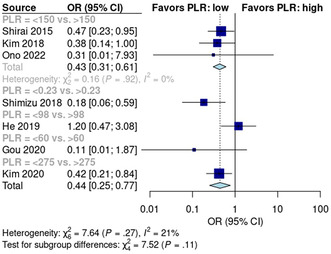

## 1 Introduction

Pancreatic ductal adenocarcinoma (PDAC) is a lethal malignancy, with a five‐year survival rate of 10%–15% [1] and surgery remains the only curative‐intent treatment. Recent technical advances, including complex vascular procedures, have increased the options for surgical resection [2]. A significant benefit in overall survival can be achieved with surgery, particularly when combined with multidisciplinary multimodal therapeutic strategies [3]. However, some studies have reported recurrence in more than 50% of resected patients in the first 12 months after surgery [4], rendering some debate of their true oncological benefit. The identification of predictive biomarkers for early recurrence would allow us to distinguish the best candidates for advanced pancreatic surgery and to optimize the treatment strategy accordingly. For instance, the subgroup of patients at higher risk of early recurrence would possibly benefit from neoadjuvant treatment strategies. These inflammatory markers could also be used to choose the optimal time of surgical exploration after neoadjuvant treatment.

Systemic immune inflammation is associated with early recurrence with consequently poor prognosis [5]. Therefore, an increasing number of studies have reported the prognostic value of inflammatory scores in PDAC. These scores include the neutrophil‐to‐lymphocyte ratio (NLR), the platelet‐to‐lymphocyte ratio (PLR), the Systemic Immune‐Inflammation Index (SII), the modified Glasgow Prognostic Score (mGPS), and the Prognostic Nutritional Index (PNI). The consensus statement by the International Study Group of Pancreatic Surgery (ISGPS) for the definition of borderline resectable PDAC also suggested that these inflammatory scores may have prognostic relevance [6]. However, results on the ability of these scores to predict recurrence are conflicting. Therefore, there is no current defined role for their use in decision‐making in PDAC.

The aim of this study was to perform a systematic review of the literature to reveal the most common inflammatory scores used to predict early recurrence after surgery for PDAC. We also performed a meta‐analysis to determine the ability of these scores to predict recurrence in patients who underwent upfront resection and those who underwent neoadjuvant chemotherapy. This could significantly impact and refine the patient selection for advanced pancreatic procedures.

## 2 Materials and Methods

This systematic review corresponds to the Preferred Reporting Items for Systematic Reviews and Meta‐Analyses (PRISMA) 2020 guidelines as well as the recommendations of the Study Centre of the German Society of Surgery [7, 8].

First, a systematic review defined the most common inflammatory scores used to predict recurrence in patients with PDAC. We included these scores in a subsequent meta‐analysis to determine their prognostic value in the rate of early recurrence after resection. Next, we evaluated how accurate these inflammatory indicators were as predictive markers for early recurrence. Finally, we performed a subgroup analysis to elucidate the predictive importance of inflammatory factors in patients who underwent upfront surgery and those who received neoadjuvant therapy.

### 2.1 Eligibility Criteria

The Population Intervention Comparison Outcome and Study design (PICOS) strategy was used to formulate the research question:

#### 2.1.1 Population

All patients undergoing curative surgery for PDAC (upfront or after neoadjuvant chemotherapy).

#### 2.1.2 Intervention

Pancreatic surgery.

#### 2.1.3 Comparator

Preoperative inflammatory scores (binary).

#### 2.1.4 Outcome

Early recurrence is defined as recurrence up to 12 months after surgery.

#### 2.1.5 Study Design

All studies except case reports, editorials, and letters to the editor.

Duplicate publications and overlapping reports were excluded.

### 2.2 Literature Search

A systematic literature search was conducted in Medline (via PubMed) and Web of Science according to the recommendations of Goossen et al. [9] using the following search terms: ((“Pancreas*” OR “Pancreat*”) AND (“Cancer*” OR “tumor*” OR “carcinom*” OR “malignan*” OR “neoplasm*”) AND (“pancreatectom*” OR “pancreatoduodenectom*” OR (“pancreas*” AND (“resection*” OR “surger*” OR “operation*” OR “surgical procedure*” OR “operative procedure*”)) AND (“recurren*” OR “Recrudescence*” OR “relapse*”) AND (“prognos*” OR “predict*” OR “prognostic factor*” OR “predictive factor*”)).

The search was restricted to studies published in or after 2000 and the last search was conducted in September 2022.

### 2.3 Study Selection and Data Extraction

Two investigators (RN and CR) conducted the primary electronic search using the predefined keywords. To identify relevant articles, they reviewed the titles and abstracts of the retrieved articles applying the inclusion and exclusion criteria. The full text of the previously identified relevant articles was screened and data were extracted by the same authors. Discrepancies were solved through discussions with the first and second authors. For each study, the following data was extracted:

#### 2.3.1 Studies Characteristics

Year of study, country of origin, and number of patients in each group (early recurrence and no early recurrence).

#### 2.3.2 Patients Characteristics

Age, gender, type of surgery (pancreaticoduodenectomy, distal pancreatectomy, total pancreatectomy), TNM stage, initial site of early recurrence, and defined inflammatory score cut‐offs reported by each study.

#### 2.3.3 Definitions and Outcomes

In mGPS, patients with elevated CRP (> 1.0 mg/dL) and hypoalbuminemia (< 3.5 g/dL) levels had a score of 2, and patients with only one of these abnormalities had a score of 1. Patients with neither of these abnormalities had a score of 0. The PNI score was calculated as 10 × serum albumin (g/dL) + 0.005 × total lymphocyte count (per mm^3^). The PLR, NLR, and SII scores were obtained from the corresponding ratios (platelet count/lymphocyte count, neutrophil count/lymphocyte count, and platelet count × neutrophil count/lymphocyte count, respectively). Early recurrence was defined as all PDAC recurrence patterns up to 12 months following pancreatic resection, according to Groot et al. [10].

### 2.4 Quality Assessment and Risk of Bias

Two investigators (RN and FB) evaluated study quality using the Risk of Bias in Non‐Randomized Studies of Interventions (ROBINS‐I) tool [11]. The overall risk of bias was considered low if the study had a low risk of bias in all categories. The study was considered to have a moderate risk of bias if there was potential bias in at least one domain, and it was assumed to have a high risk of bias if there was a high risk of bias in at least one domain or some concerns of bias in many domains. Grading of Recommendations, Assessment, Development, and Evaluation (GRADE) was used to assess the quality of the evidence for each outcome [12].

### 2.5 Statistical Analysis

To assess the correlation between preoperative inflammatory scores and early recurrence, pooled odds ratios (ORs) and 95% confidence intervals were computed using a random effects model of meta‐analysis. To compare the diagnostic accuracy of the markers, a summary receiver operating characteristic (SROC) curve was obtained to determine their overall predictive power. Statistical heterogeneity was calculated using the *χ* ^2^ test and inconsistency analysis, and heterogeneity was considered significant if the *p* < 0.05 and the *I* ^2^ value was larger than 50%. Publication bias was evaluated using visual inspection funnel plots and the Egger test. In all analyses, *p* < 0.05 were considered significant. R language [13], R studio [14], and R packages (Mada [15] and Meta [16]) were used to conduct the statistical analysis.

## 3 Results

### 3.1 Literature Search

**FIGURE 1:**
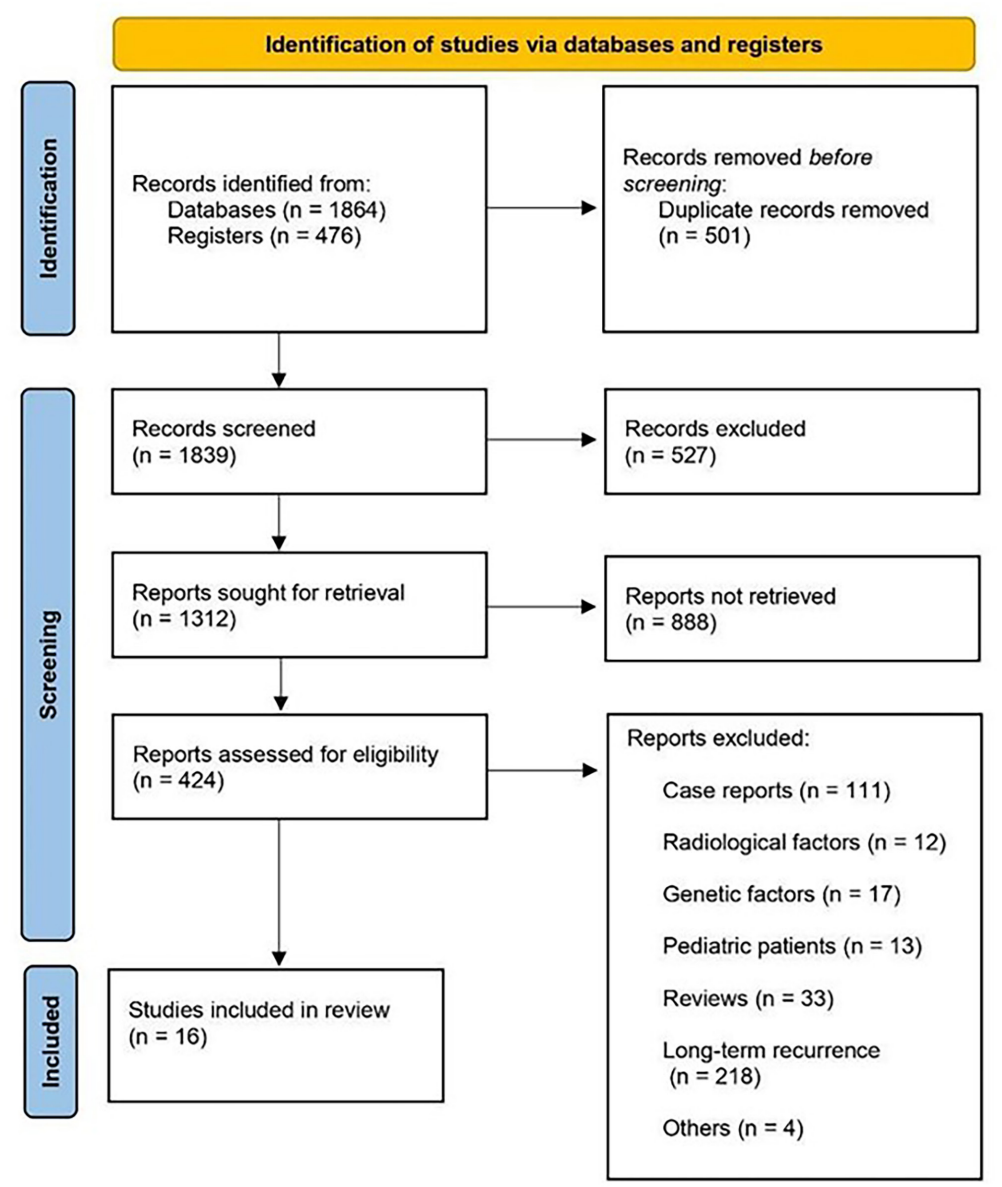
PRISMA flowchart showing a selection of articles for review.

Our systematic search identified 2340 articles. After duplicates were removed, the titles and abstracts of 1839 studies were reviewed for inclusion criteria and 424 articles were included in the full‐text review. In the end, 16 studies were included in the meta‐analysis (Figure 1). Nine studies reported outcomes for mGPS, four studies for PNI, seven studies for PLR, eight studies for NLR, and two studies for SSI. The baseline characteristics of these studies, which included 4460 patients, are summarized in Table 1.

**TABLE 1:** Characteristics of included studies in the current meta‐analysis.

Study (year)	Study design	Country	Group	Age (mean ± SD)	Gender (M/F)	BMI (mean ± SD)	Type of operation	TNM stage	Early recurrence (months)	Recurrence site
Zou et al. (2022) [17]	Retrospective	China	No early recurrence: 457/976 (46.8%)	62.26 ± 8.89	272/185	24.6 ± 3.5	Pancreaticoduodenectomy: 303 (66%) Distal pancreatectomy: 137 (30%) Total pancreatectomy: 30 (4%)	≤ 2A: 253 (55%) > 2A: 204 (45%)	2–12	Local: 61/199 (30.7%) Systemic: 81/199 (40.7%) Multiple: 22/199 (11.1%) Unknown: 35/199 (17.6%)
			Early recurrence: 519/976 (53.2%)	62.75 ± 8.93	327/192	25.4 ± 5.6	Pancreaticoduodenectomy: 335 (66%) Distal pancreatectomy: 154 (28%) Total pancreatectomy: 30 (6%)	≤ 2A: 285 (55%) > 2A: 234 (45%)		Local: 82 (15.8%) Systemic: 309 (59.5%) Multiple: 62 (12%) Unknown: 66 (12.7%)
Ono et al. (2022) [18]	Retrospective	Japan	No early recurrence: 32/66 (48.5%)	64.5 (45–76)	13/19	NA	NA	≤ 2A: 18 (56.2%) > 2A: 14 (43.8%)	6–12	NA
			Early recurrence: 34/66 (51.5%)	69.0 (44–84)	19/15	NA	NA	≤ 2A: 5 (14.7%) > 2A: 29 (85.3%)		NA
Sugawara et al. (2021) [19]	Retrospective	Japan	No early recurrence: 101/136 (74.3%)	67.5 ± 2.67	61/40	NA	NA	NA	6	NA
			Early Recurrence: 35/136 (25.7%)	72 ± 3.25	21/14	NA	NA	NA		NA
Kim et al. (2020) [20]	Retrospective	South Korea	No early recurrence: 299/631 (47.3%)	62.8 ± 10.3	176/ 123	23 ± 3	NA	NA	12	NA
			Early recurrence: 332/631 (52.7%)	63.2 ± 10.1	201/131	22.9 ± 3.1	NA	NA		Local: 68 (20.4%) Systemic: 265 (79.6%)
Gou et al. (2020) [21]	Retrospective	China	No early recurrence: 235/324 (72.5%)	NA	149/86	NA	NA	NA	5	Local: 55/146 (37.7%) Systemic: 66/146 (45.2%) Multiple: 25/146 (17.1%)
			Early recurrence: 89/324 (27.5%)	NA	52/37	NA	NA	NA		Local: 12 (13.4%) Systemic: 41 (46%) Multiple: 36 (40.6%)
Tsuchiya et al. (2019) [22]	Retrospective	Japan	No early recurrence: 31/61 (50.8%)	67.2 ± 7.94	18/13	NA	NA	NA	12	Local: 2/11 (18.2%) Systemic: 9/11 (81.8%)
			Early recurrence: 30/61 (49.2%)	63.8 ± 9.08	17/13	NA	NA	NA		Local: 3 (10%) Systemic: 27 (90%)
Suto et al. (2019) [23]	Retrospective	Japan	No early recurrence: 81/115 (70.4%)	NA	43/38	NA	NA	NA	6	NA
			Early recurrence: 34/115 (29.6%)	NA	19/15	NA	NA	NA		NA
He et al. (2019) [24]	Retrospective	China	No early recurrence: 258/302 (85.4%)	NA	159/99	NA	Pancreaticoduodenectomy or Distal pancreatectomy	≤ 2A: 135 (52.3%) > 2A: 123 (47.7%)	12	NA
			Early recurrence: 44/302 (14.6%)	NA	24/20	NA		≤ 2A: 28 (63.6%) > 2A: 16 (36.4%)		NA
Aziz et al. (2019) [25]	Retrospective	Netherlands	No early recurrence: 157/295 (53.2%)	NA	NA	NA	NA	NA	12	NA
			Early recurrence: 138/295 (46.8%)	NA	NA	NA	NA	NA		NA
Shimizu et al. (2018) [26]	Retrospective	Japan	No early recurrence: 62/84 (73.8%)	69 ± 7	37/25	NA	Pancreaticoduodenectomy or Distal pancreatectomy	NA	6	NA
			Early recurrence: 22/84 (26.2%)	65 ± 8.6	15/7	NA		NA		NA
Kim et al. (2018) [27]	Retrospective	South Korea	No early recurrence: 55/81 (67.9%)	70.2 ± 8.6	35/20	23.0 ± 3.3	Pancreaticoduodenectomy: 38 (69.1%) Distal pancreatectomy: 16 (29.1%) Total pancreatectomy: 1 (1.8%)	≤ 2A: 31 (56.3%) > 2A: 24 (43.7%)	6	NA
			Early recurrence: 26/81 (32.1%)	70.2 ± 9.9	17/9	22.8 ± 3.3	Pancreaticoduodenectomy: 21 (80.7%) Distal pancreatectomy: 5 (19.3%) Total pancreatectomy: 0	≤ 2A: 8 (30.7%) > 2A: 18 (69.3%)		NA
Nishio et al. (2017) [28]	Retrospective	Japan	No early recurrence: 58/90 (64.4%)	NA	25/33	NA	Pancreaticoduodenectomy: 41 (45.5%) Distal pancreatectomy: 47 (52.2%) Total pancreatectomy: 2 (2.3%)	≤ 2A: 48 (53.3%) > 2A: 42 (46.7%)	12	Local: 22/28 (78.6%) Systemic: 6/28 (21.4%)
			Early recurrence: 32/90 (35.6%)	NA	17/15	NA				Local: 10 (31.2%) Systemic: 22 (68.8%)
Asaoka et al. (2016) [29]	Retrospective	Japan	No early recurrence: 14/46 (30.4%)	67 ± 8.9	22/24	23 ± 3.1	NA	NA	12	NA
			Early recurrence: 32/46 (69.6%)				NA	NA		NA
Shirai et al. (2015) [30]	Retrospective	Japan	No early recurrence: 70/131 (53.4%)	66.5 ± 10.2	81/50	NA	Pancreaticoduodenectomy: 87 (66.5%) Distal pancreatectomy: 41 (31.2%) Total pancreatectomy: 3 (2.3%)	NA	12	NA
			Early recurrence: 61/131 (46.6%)			NA		NA		NA
Matsumoto et al. (2015) [31]	Retrospective	Japan	No early recurrence: 729/968 (75.3%)	67 ± 10	404/325	21.7 ± 3.1	Pancreaticoduodenectomy: 483 (66%) Distal pancreatectomy: 230 (32%) Total pancreatectomy: 16 (2%)	≤ 2A: 94 (13%) > 2A: 635 (87%)	6	NA
			Early recurrence: 239/968 (24.7%)	67 ± 10	131/108	21.3 ± 3	Pancreaticoduodenectomy: 158 (66%) Distal pancreatectomy: 68 (29%) Total pancreatectomy: 13 (5%)	≤ 2A: 11 (5%) > 2A: 228 (95%)		NA
Sugiura et al. (2012) [32]	Retrospective	Japan	No early recurrence: 106/154 (68.8%)	NA	65/41	NA	Pancreaticoduodenectomy: 114 (74%) Distal pancreatectomy: 38 (24.6%) Total pancreatectomy: 2 (1.4%)	NA	6	Local: 13/65 (20%) Systemic: 52/65 (80%)
			Early recurrence: 48/154 (31.2%)	NA	30/18	NA		NA		Local: 13 (27%) Systemic: 35 (73%)

### 3.2 Risk of Bias Assessment

The articles included in this review were published between 2012 and 2022 and all were retrospective. Of these 16 studies, five had a serious risk of bias, six had a moderate risk of bias, and five had a low risk of bias (Table 2).

**TABLE 2:** Quality assessment of the included studies.

Study	Risk of bias							
	Bias due to confounding	Bias in the selection of participants for the study	Bias in the classification of interventions	Bias due to deviations from intended interventions	Bias due to missing data	Bias in the measurement of outcomes	Bias in the selection of the reported result	ROBINS‐I (overall)
Zou et al. (2022) [17]	Low	Low	Low	Low	Low	Low	Low	Low
Ono et al. (2022) [18]	Serious	Low	Low	Low	Moderate	Moderate	Low	Serious
Sugawara et al. (2021) [19]	Serious	Low	Low	Low	Moderate	Low	Moderate	Serious
Kim et al. (2020) [27]	Serious	Moderate	Low	Low	Moderate	Serious	Moderate	Serious
Gou et al. (2020) [21]	Moderate	Low	Low	Low	Moderate	Low	Moderate	Moderate
Tsuchiya et al. (2019) [22]	Low	Low	Low	Low	Low	Low	Low	Low
Suto Et al. (2019) [23]	Moderate	Moderate	Low	Moderate	Moderate	Low	Moderate	Serious
He et al. (2019) [24]	Moderate	Moderate	Low	Low	Moderate	Low	Moderate	Moderate
Aziz et al. (2019) [25]	Moderate	Low	Low	Low	Moderate	Low	Moderate	Moderate
Shimizu et al. (2018) [26]	Serious	Low	Low	Low	Moderate	Low	Moderate	Serious
Kim et al. (2018) [27]	Moderate	Low	Moderate	Low	Moderate	Low	Moderate	Moderate
Nishio et al. (2017) [28]	Low	Low	Low	Low	Low	Low	Low	Low
Asaoka et al. (2016) [29]	Low	Low	Low	Low	Moderate	Moderate	Moderate	Low
Shirai et al. (2015) [30]	Moderate	Low	Low	Low	Moderate	Low	Low	Moderate
Matsumoto et al. (2015) [31]	Low	Low	Low	Low	Low	Low	Low	Low
Sugiura et al. (2012) [32]	Moderate	Low	Low	Moderate	Low	Low	Low	Moderate

### 3.3 Pooled Rate of Early Recurrence

A random effects model of meta‐analysis showed a pooled recurrence rate of 37% [95% confidence interval (CI): 30%, 46%] among the included studies (Figure S1).

### 3.4 Predictive Role of mGPS for Early Recurrence

**FIGURE 2:**
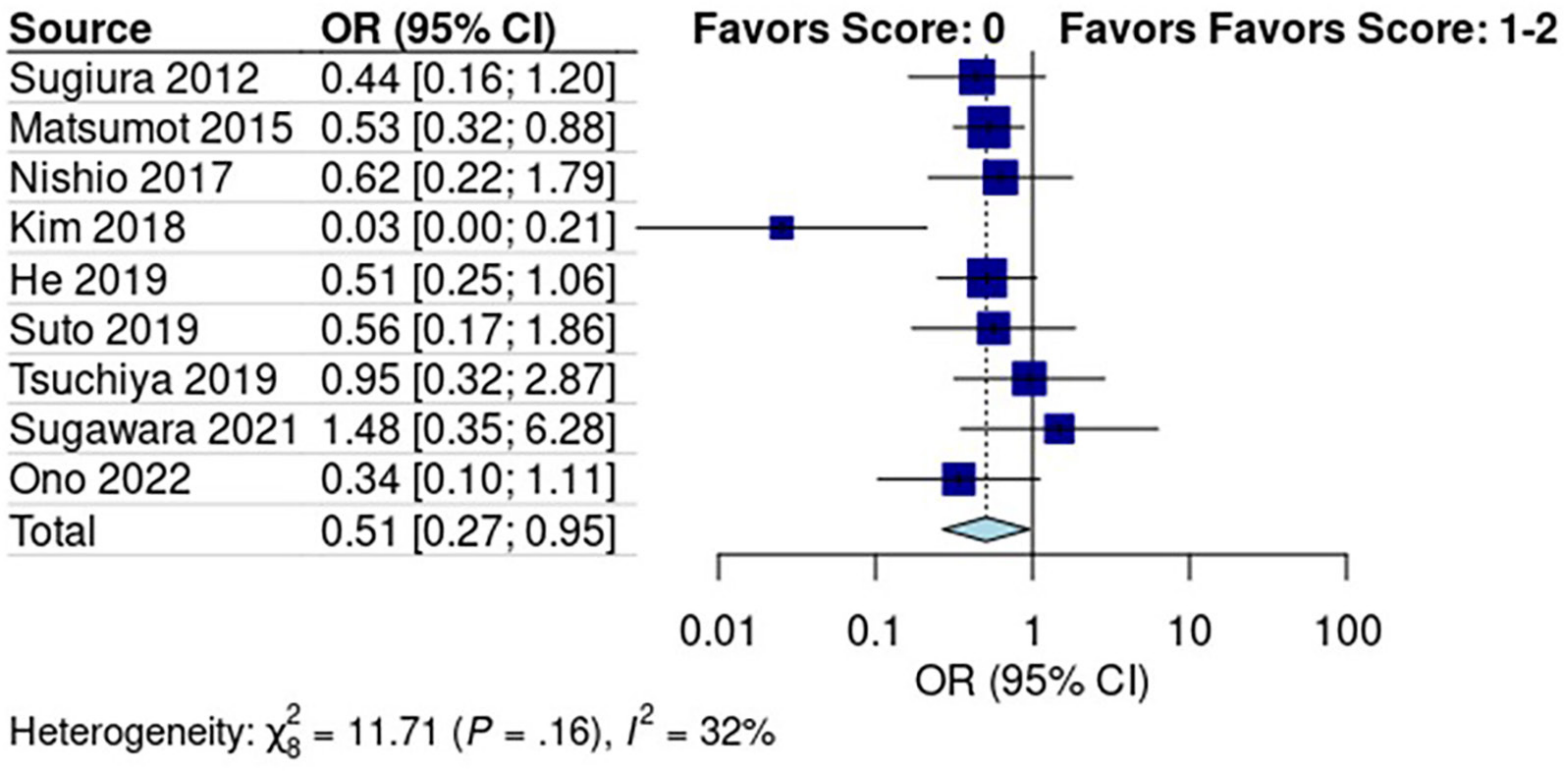
(A) Predictive role of (A) Modified Glasgow Prognostic Score, and (B) Prognostic Nutritional Index in early recurrence after pancreatectomy.

Subgroup analysis of the studies reporting mGPS scores revealed a pooled recurrence rate of 30% [95% CI: 0.22, 0.40] in the low score group (mGPS score: 0) and 46% [95% CI: 0.28, 0.65] in the high score group (mGPS score: 1–2) (Figure S2). The Mantel–Haenszel test showed that early recurrence was significantly lower in the low‐score group than in the high‐score group (*p* < 0.01, OR: 0.51, 95% CI: 0.27, 0.95; Figure 2). Pooled studies had low heterogeneity (*I* ^2^ = 32%; *p* = 0.16).

### 3.5 Predictive Role of PNI for Early Recurrence

**FIGURE 3:**
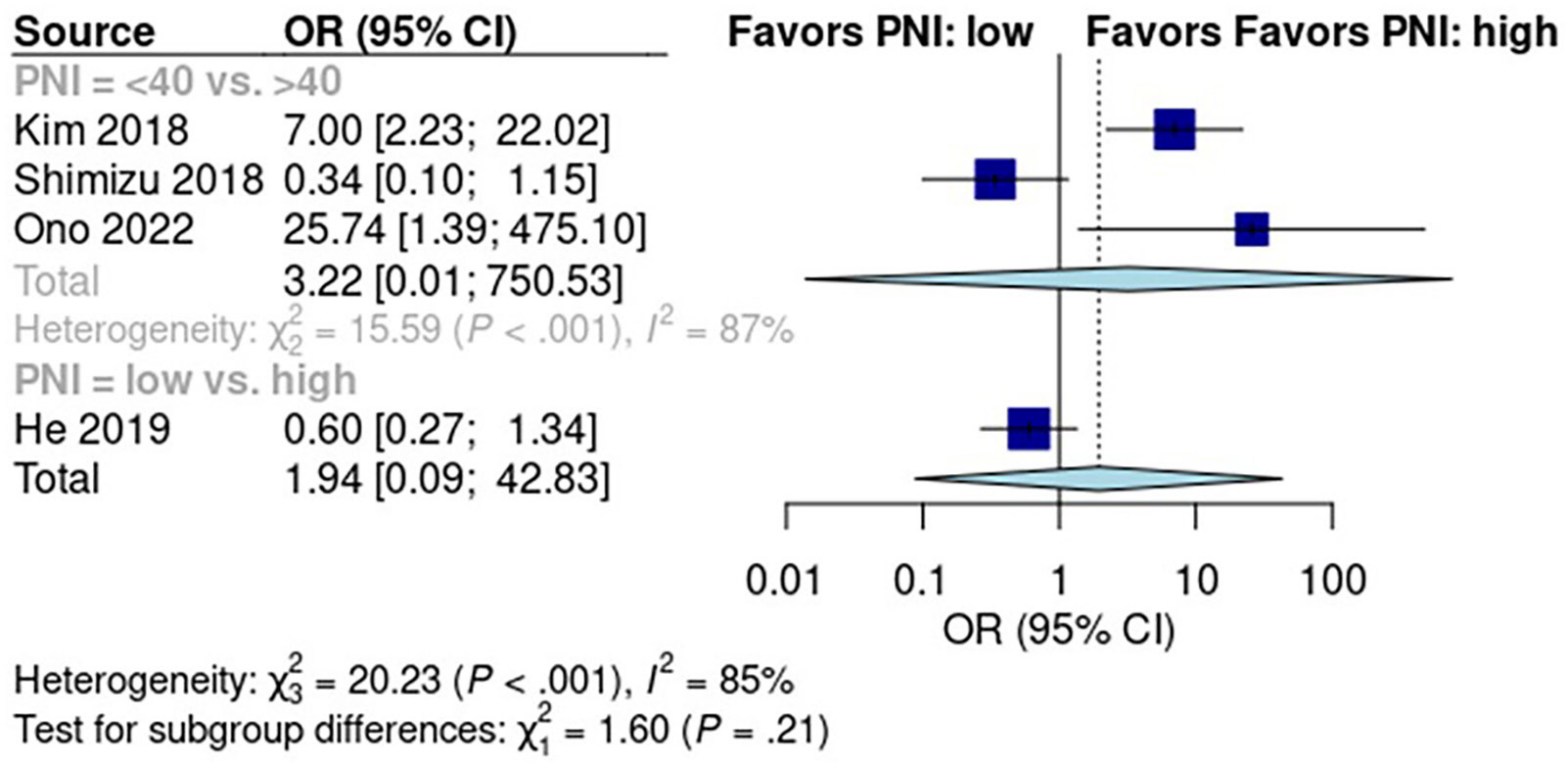
(A) Predictive role of (A) platelet to lymphocyte ratio, (B) neutrophil to lymphocyte ratio, and (C) Systemic‐Immune‐Inflammation Index in early recurrence after pancreatectomy.

Subgroup analysis of studies reporting PNI scores revealed a pooled early recurrence rate of 52% [95% CI: 3%, 97%] in the low‐score group and 27% [95% CI: 14%, 47%] in the high‐score group (Figure S3). A random effects model showed no significant difference in early recurrence between the low PNI score and high PNI score groups (*p* > 0.05; OR: 1.94; 95% CI: 0.09, 42.83; Figure 3). Data from the five pooled studies showed high heterogeneity (*I* ^2^ = 85%; *p* = 0.21).

### 3.6 Predictive Role of PLR for Early Recurrence

**FIGURE 4:**
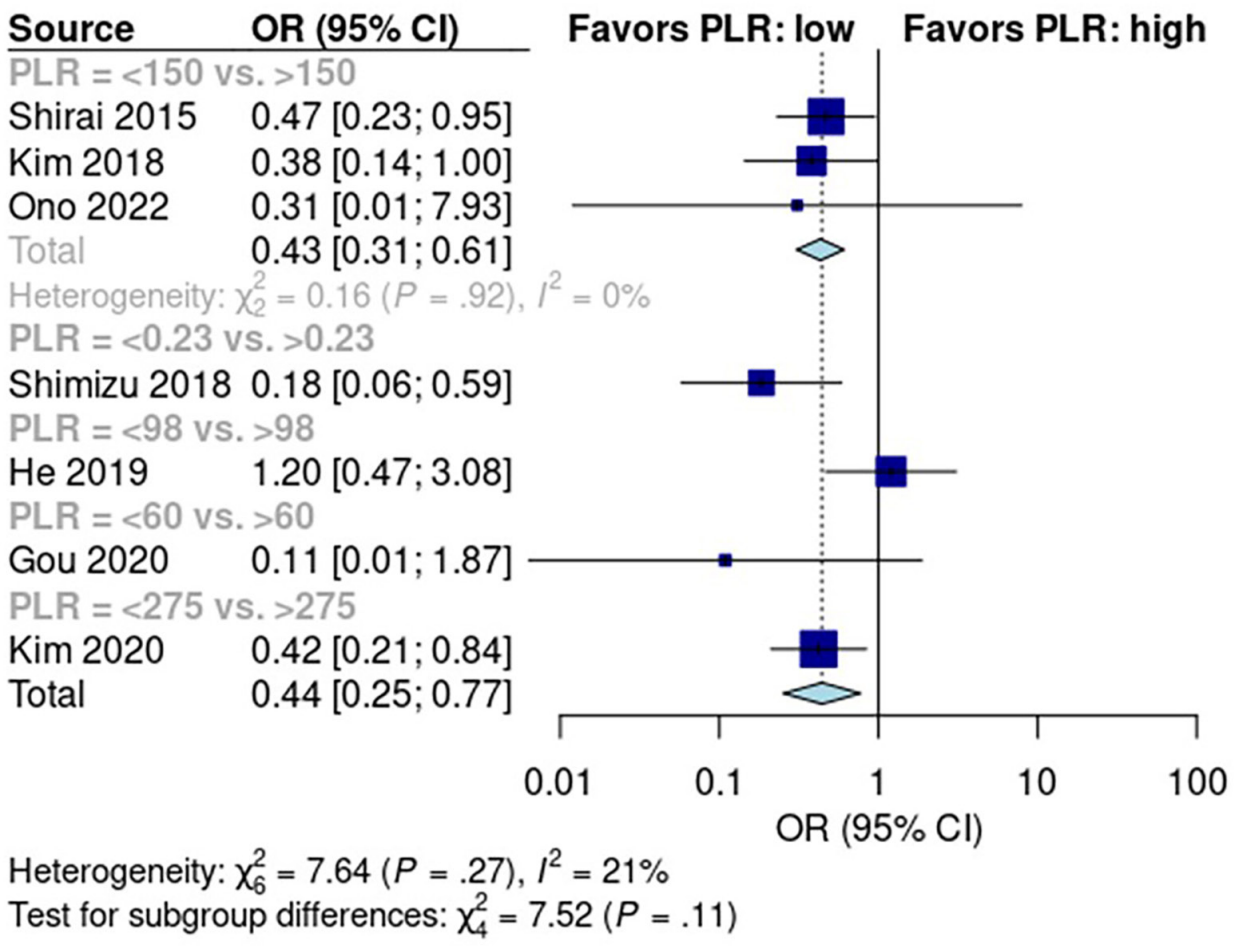
Comparison of the diagnostic accuracy (SROCs) of PLR, NLR, and mGPS in predicting early recurrence after pancreatic resection. PLR, AUC = 0.607; NLR, AUC = 0.628; mGPS, AUC = 0.534.

The pooled early recurrence rate was 28% [95% CI: 15%, 46%] in patients with low PLR scores and 44% [95% CI: 24%, 66%] in patients with high PLR scores (Figure S4). Random effects analysis showed that the early recurrence rate was significantly higher in the high PLR score group than in the low PLR score group (*p* < 0.01; OR: 0.44; 95% CI: 0.25, 0.77; Figure 4). Data from the six pooled studies showed no significant heterogeneity (*I* ^2^ = 21%; *p* = 0.27).

### 3.7 Predictive Role of NLR for Early Recurrence

**FIGURE 5:**
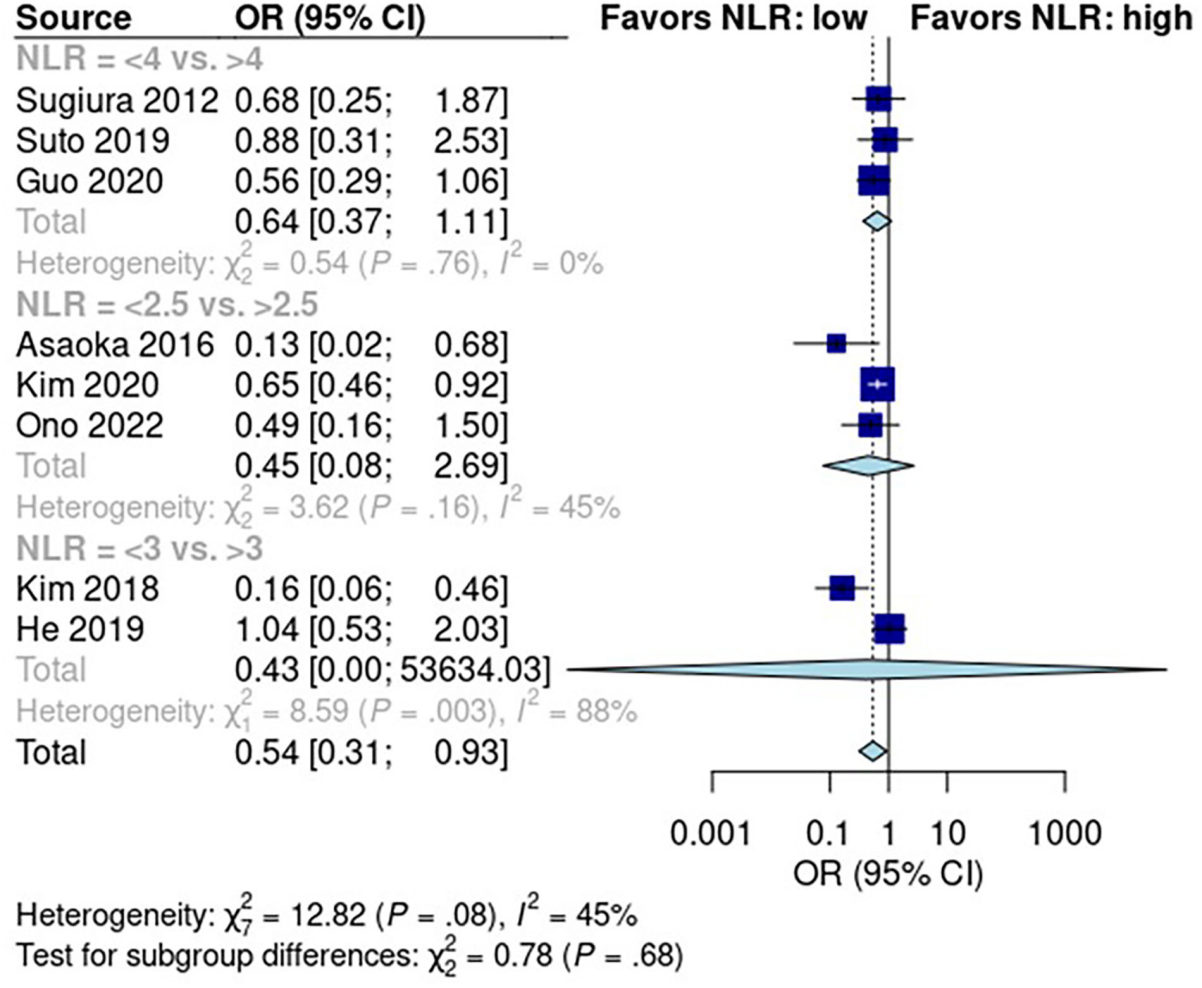
Predictive role of neutrophil to lymphocyte ratio in early recurrence after pancreatectomy.

NLR was reported in eight studies with 1976 patients—1225 in the low NLR score group and 751 in the high NLR score group. The pooled early recurrence rate was 32% [95% CI: 22%, 45%] in patients with low NLR scores and 50% [95% CI: 30%, 70%] in patients with high NLR scores (Figure S5). A random effects model revealed that the rate of early recurrence was significantly higher in patients with high NLR scores than in patients with low NLR scores (*p* < 0.05; OR: 0.54; 95% CI: 0.31, 0.93; Figure 5). Pooled studies had low heterogeneity (*I* ^2^ = 45%; *p* = 0.08).

### 3.8 Predictive Role of SII for Early Recurrence

**FIGURE 6:**
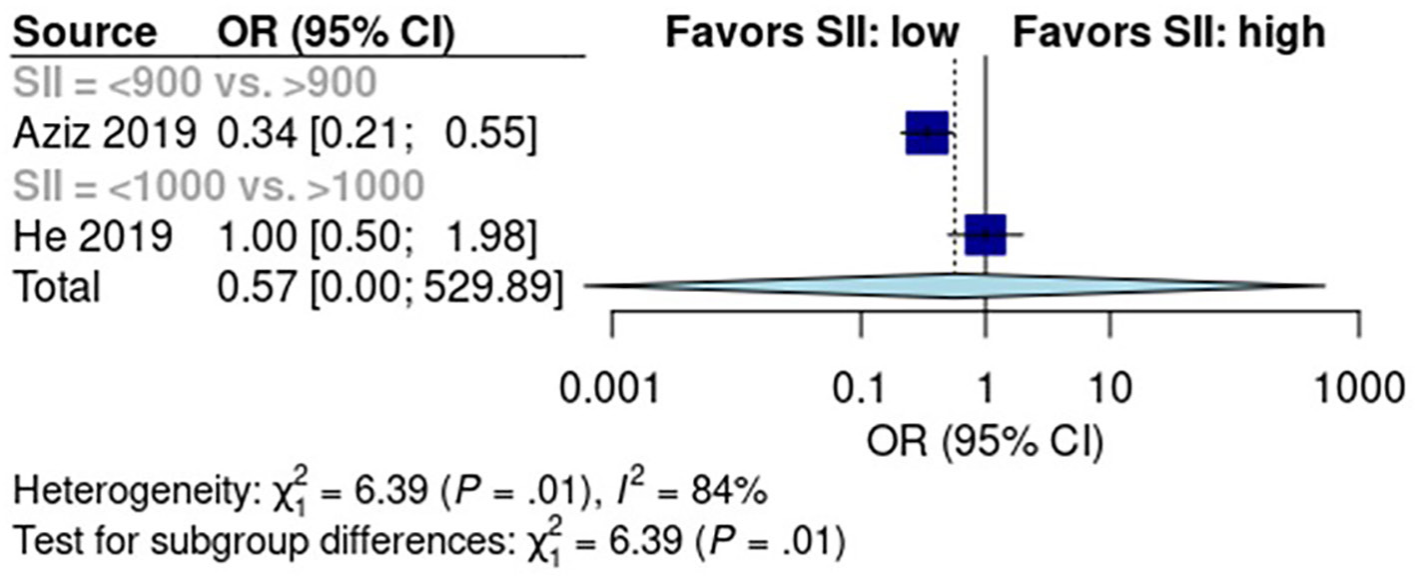
Predictive role of Systemic‐Immune‐Inflammation Index in early recurrence after pancreatectomy.

Two studies reported SII scores in 597 patients—366 had low SII scores and 231 had high SII scores. The pooled early recurrence rate was 26% [95% CI: 0%, 100%] in the low SII score group and 37% [95% CI: 0%, 100%] in the high SII score group (Figure S6). Mantel–Haenszel analysis showed no difference in early recurrence rate between low‐ and high SII score groups (*p* > 0.05, Figure 6). Data from the two included studies had high heterogeneity (*I* ^2^ = 84%, *p* < 0.01).

### 3.9 Diagnostic Accuracy of the Inflammatory Markers

**FIGURE 7:**
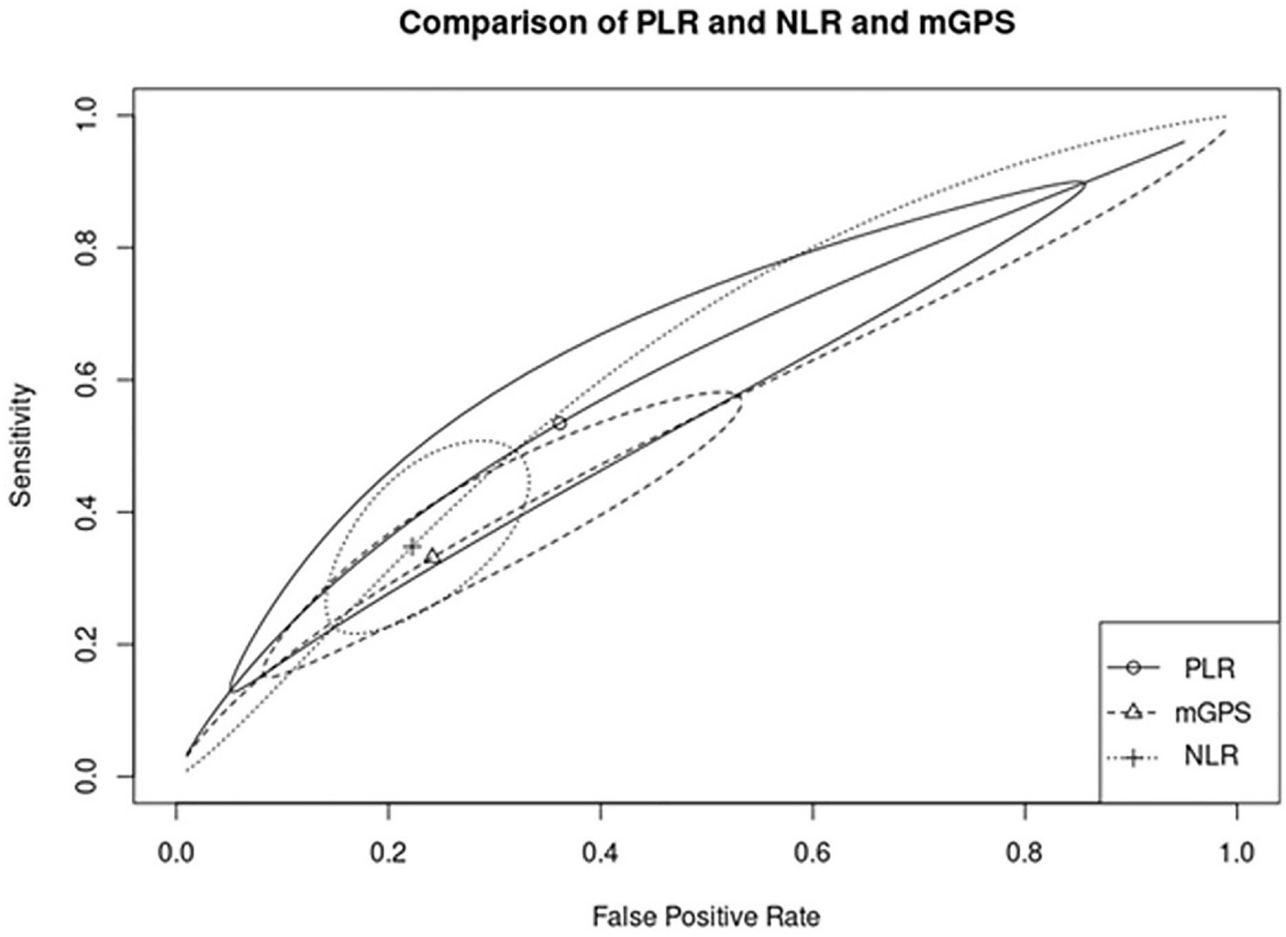
Comparison of the diagnostic accuracy (SROCs) of PLR, NLR, and mGPS in predicting early recurrence after pancreatic resection. PLR, AUC = 0.607; NLR, AUC = 0.628; mGPS, AUC = 0.534.

The diagnostic accuracy of inflammatory markers was compared between markers shown to have a significant prognostic role in the first part of the analysis (mGPS, NLR, and PLR). To reach this goal, a SROC curve was used to compare the overall discriminatory ability of the inflammatory scores. No significant difference was found in the predictive accuracy of mGPS (pooled sensitivity: 33.1%; 95% CI: 17.8%, 53.1%; pooled specificity: 75.9%; 95% CI: 53.1%, 89.7%; AUC = 0.534), NLR (pooled sensitivity: 34.8%, 95% CI: 23.9%, 47.5%; pooled specificity: 77.8%; 95% CI: 69.2%, 84.5%; AUC = 0.628), and PLR (pooled sensitivity: 53.4%, 95% CI: 18.1%, 85.6%; pooled specificity: 63.9%, 95% CI: 21.1%, 92.1%; AUC = 0.607), as shown in Figure 7.

### 3.10 Subgroup Analysis According to Preoperative Treatment

In the final analysis, studies were divided according to preoperative therapy into an upfront resection group and neoadjuvant chemotherapy with a resection group. In the upfront resection group, high mGPS and PLR scores were associated with early recurrence (mGPS: *p* < 0.05; OR: 0.54; 95% CI: 0.39, 0.75, Figure S7 and PLR: *p* < 0.05; OR: 0.44; 95% CI: 0.22, 0.86, Figure S9). On the other hand, PNI and NLR scores had no significant effect on the risk of early recurrence (*p* > 0.05, Figures S8 and S10, respectively). In the neoadjuvant chemotherapy with resection group, a low PNI score increased the rate of early recurrence (Figure S8) whereas mGPS, PLR, and NLR scores had no significant effect on the risk of early recurrence (Figures S7, S9 and S10, respectively). We could not perform a subgroup analysis for SII as the studies did not report the preoperative treatment.

### 3.11 Quality of Evidence Assessment

The quality of evidence was very low according to the GRADE assessment (Table 3).

**TABLE 3:** Grades of Recommendation, Assessment, Development, and Evaluation (GRADE) assessment for outcomes of interest.

Comparison	Studies	GRADE							
		Risk of bias	Inconsistency	Indirectness	Imprecision	Publication bias	Large or moderate effect size	Exposure‐response gradient	Overall quality
*Low score versus. high score*									
Modified Glasgow Prognostic Score	9	Very serious	Serious	Not serious	Serious	Not serious	Not present	Not present	Very low
Prognostic Nutritional Index	4	Very serious	Serious	Not serious	Not serious	Not serious	Not present	Not present	Very low
Platelet to lymphocyte ratio	7	Serious	Serious	Not serious	Serious	Serious	Not present	Not present	Very low
Neutrophil to lymphocyte ratio	8	Serious	Not serious	Not serious	Serious	Not serious	Not present	Not present	Very low
Systemic‐Immune‐Inflammation Index	2	Very serious	Not serious	Serious	Serious	Not serious	Not present	Not present	Very low

## 4 Discussion

In recent years, pancreatic surgery has seen many technical advances such as artery and uncinated‐first approaches, dissection of the anatomical triangle between the coeliac and superior mesenteric arteries and the portomesenteric vein, complex procedures for resection and reconstruction of the mesenteric–portal vein axis, arterial divestments, and even arterial resection [2, 33, 34]. These advanced techniques are used in multimodal interdisciplinary treatment strategies, including good patient selection (staging), multi‐agent neo and adjuvant chemotherapy regimens, complication management, and enhanced recovery programs. This has improved the outcomes, safety, and efficacy of pancreatic surgery, expanding surgical indications to locally advanced tumors [3]. However, more than 50% of patients with PDAC experience recurrence in the first 12 months after surgery [4]. Screening out and identifying patients at a higher risk of early recurrence would allow treatment to be individualized to improve the prognosis and survival. Unfortunately, there is no current structured model in clinical practice to effectively predict early recurrence or prognosis of patients with PDAC undergoing surgical resection.

There is solid evidence that systemic immune inflammation is related to poor prognosis in oncological patients [35, 36]. Scores that measure the inflammatory activity of neutrophils, lymphocytes, and platelets (such as the NLR, PLR, SII, and mGPS) have been increasingly used to predict the prognosis of patients, including PDAC [37]. In the present study, we revealed significant differences in early recurrence rates between groups with high and low mGPS, NLR, and PLR scores. These findings are in line with those of previous studies suggesting that CRP, neutrophils, and platelets are risk factors for a worse prognosis, while lymphocytes are considered protective [38, 39, 40]. In contrast, we found that PNI and SII scores were not correlated with early recurrence rates.

Although our pooled analysis showed significant differences in early recurrence for mGPS, NLR, and PLR scores, the diagnostic accuracy of these scores for early recurrence was low (AUCs of 0.534, 0.628, and 0.607, respectively). This suggests that they are not capable of supporting clinical and oncological decisions. There are several possible explanations for these results. First, there was high heterogeneity in the application of these scores between studies. For example, the reported cut‐offs for the different ratios were different between all included studies [17, 32] and the timing of measurement was also unclear. Compared to the ratio‐based scores with their poorly defined cut‐offs, a score like mGPS with its well‐defined values may have an advantage in standard clinical application. Second, many factors affect immune response such as infection, therapeutical regimens (such as neoadjuvant chemotherapy), or nutritional status that were not possible to take into consideration in our analysis. Lastly, there is also some evidence reporting that the use of these inflammatory markers is dependent on bilirubin levels [25], but these data were not available. Ultimately, PDAC progression likely depends on an intricate and complex interplay between tumor‐bearing host factors, such as systemic immune inflammation, and the tumor microenvironment [41, 42].

Despite their low discriminative ability, mGPS and NLR scores showed good specificity (75.9% and 77.8%, respectively) for predicting early recurrence. This suggests that these scores could be combined with other highly sensitive prognostic factors to predict early recurrence in a stepwise approach, nomograms, or multimarker panels, possibly resulting in satisfactory prognostic accuracy. Ca 19‐9, as the most extensively evaluated biomarker with a reported sensitivity of 79% [43], could be a logical candidate. Furthermore, Ca 19‐9 has been shown to relate to early recurrence. In our analysis, all studies reporting Ca 19‐9 showed that it had similar or better diagnostic accuracy than inflammatory scores did [10, 17, 32]. Other clinical, radiological, histopathological, and genetic prognostic factors should also be considered [44].

We also performed a subgroup analysis regarding the preoperative treatment strategy. As previously mentioned, the increase in “resectability” with recent technical advances has resulted in a trend away from anatomical definitions to a biology‐based approach to PDAC treatment [3, 6]. The subgroup of patients with a worse prognosis and higher risk of early recurrence would possibly benefit from neoadjuvant systemic therapy. Also in patients under neoadjuvant regimens, this prognostic evaluation would aid in choosing short or long chemotherapy courses or even the optimal timing for surgical exploration. Unfortunately, the inflammatory markers analyzed in our study were not accurate and reliable enough to support these decisions.

In this study, the pooled rate of early recurrence among the included studies was 37%. This is lower than most studies reporting this outcome [4]. This could be explained by the definitions of early recurrence. According to the definition of Groot et al. [10], we defined early recurrence as any type of recurrence occurring in the first 12 months after pancreatic resection as this time period showed to correlate best with the post‐recurrence‐free survival and prognosis. However, several studies included in our analysis used shorter time periods, likely resulting in an underestimation of the early recurrence rate.

There are limitations to the present study. The main weakness is the retrospective nature of the included studies. This need for better evidence was also recognized in a recent consensus statement on mandatory measurements in pancreatic cancer trials [45]. Another limitation is that the majority of the included studies in the meta‐analysis are based on East Asia populations. Further studies concerning the Western population are needed to clarify any regional differences in prognosis related to the inflammatory markers. A further limitation is that data on the outcomes of interest were not available in all studies, which reduced the power of the analysis. Additionally, we were not able to carry out subgroup analyses for the presence of jaundice or infectious/inflammatory conditions, type of pancreatic resection, staging, histopathological features, or other factors that may significantly affect prognosis.

In conclusion, mGPS, PNI, PLR, NLR, and SII scores did not show suitable diagnostic accuracy to predict recurrence in the first 12 months after pancreatic resection for PDAC. Therefore, these inflammatory scores are not helpful in selecting the best surgical candidates. Further higher quality studies are needed to comprehensively evaluate the prognostic value of the inflammatory scores, possibly in combination with other clinical, radiological, or genetic predictive factors.

## Author Contributions

**Filipe de Castro e Borges:** conceptualization (equal), data curation (equal), investigation (equal), methodology (equal), supervision (equal), validation (equal), writing – original draft (equal), writing – review and editing (equal). **Elias Khajeh:** data curation (equal), formal analysis (equal), investigation (equal), methodology (equal), validation (equal), visualization (equal), writing – original draft (equal), writing – review and editing (equal). **Rajan Nikbakhsh:** data curation (equal), formal analysis (equal), methodology (equal), validation (equal), visualization (equal), writing – original draft (equal), writing – review and editing (equal). **Catarina Ribeiro:** data curation (equal), formal analysis (equal), investigation (equal), validation (equal), writing – original draft (equal), writing – review and editing (equal). **Markus Maeurer:** data curation (equal), investigation (equal), methodology (equal), validation (equal), writing – review and editing (equal). **Carlos Carvalho:** data curation (equal), investigation (equal), methodology (equal), validation (equal), writing – review and editing (equal). **Gil Gonçalves:** data curation (equal), investigation (equal), methodology (equal), validation (equal), writing – review and editing (equal). **Christoph Berchtold:** data curation (equal), investigation (equal), methodology (equal), validation (equal), writing – review and editing (equal). **Markus W. Büchler:** investigation (equal), methodology (equal), supervision (equal), validation (equal), writing – review and editing (equal). **Arianeb Mehrabi:** conceptualization (equal), data curation (equal), investigation (equal), methodology (equal), project administration (equal), supervision (equal), validation (equal), writing – review and editing (equal).

## Ethics Statement

The authors have nothing to report.

## Consent

The authors have nothing to report.

## Conflicts of Interest

The authors declare no conflicts of interest.

## Supporting information


Data S1.

## Data Availability

The data used for this study can be made available upon request to the corresponding author.
