# Supplementary material for: Preoperative Inflammatory Scores Do Not Accurately Predict Early Recurrence of Pancreatic Ductal Adenocarcinoma After Resection: A Systematic Review and Meta‐Analysis
Source: Cancer Med. 2024 Oct 29;13(20):e70352. doi: 10.1002/cam4.70352 (PMC11519998; doi:10.1002/cam4.70352)

**Supplementary Material**

**Preoperative Inflammatory Scores Are Not Helpful to Predict Early Recurrence of Pancreatic Ductal Adenocarcinoma After Resection: A Systematic Review and Meta-Analysis**

Filipe de Castro e Borges^1, 2^; Elias Khajeh^1,3^; Rajan Nikbakhsh^1,3^; Catarina Ribeiro^2^;

Markus Maeurer^4^; Carlos Carvalho^5^; Gil Gonçalves^1^; Christoph Berchtold^3^;

Markus W. Büchler^3^; Arianeb Mehrabi^3^

1. Department of Digestive Surgery, Hepato-Pancreato-Biliary Surgery Unit, Champalimaud Foundation; Lisbon, Portugal
2. Faculdade de Medicina, Universidade de Lisboa; Lisbon, Portugal
3. Department of General, Visceral and Transplantation Surgery, University Hospital Heidelberg, Im Neuenheimer Feld 420, 69120 Heidelberg, Germany
4. Champalimaud Centre for the Unknown; Lisbon, Portugal
5. Digestive Unit, Clinical Oncology, Champalimaud Clinical Centre; Lisboa, Portugal.

**Correspondence:**

Professor Dr. Arianeb Mehrabi FICS, FEBS, FACS

Head of Division of Liver Surgery and Abdominal Transplantation

Department of General, Visceral and Transplantation Surgery

Heidelberg University Hospital

Im Neuenheimer Feld 420, 69120, Heidelberg, Germany

E-mail: Arianeb.Mehrabi@med.uni-heidelberg.de

**Supplementary Figure 1**. Pooled rate of early recurrence.


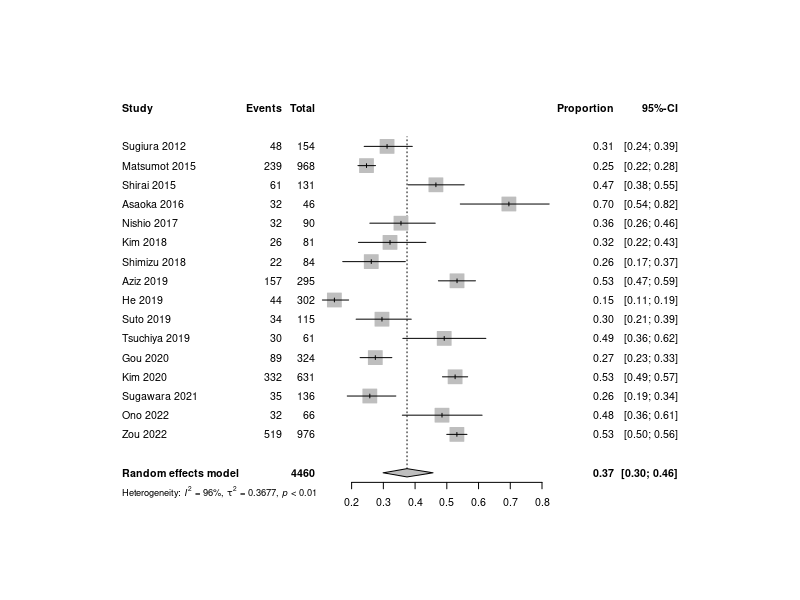


**Supplementary Figure 2.** Subgroup analysis of pooled rate of early recurrence based on Modified Glasgow Prognostic Score


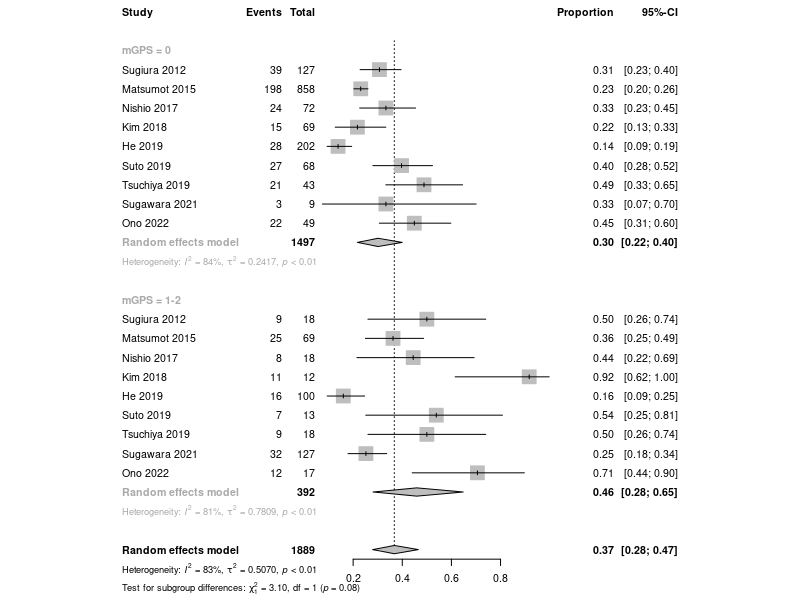


**Supplementary Figure 3.** Subgroup analysis of pooled rate of early recurrence based on Prognostic Nutritional Index


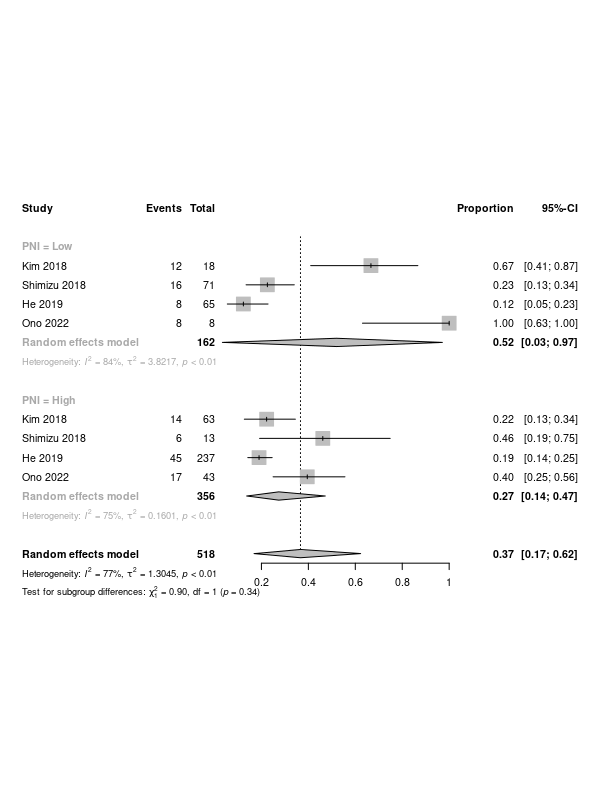


**Supplementary Figure 4.** Subgroup analysis of pooled rate of early recurrence based on Platelet to Lymphocyte Ratio


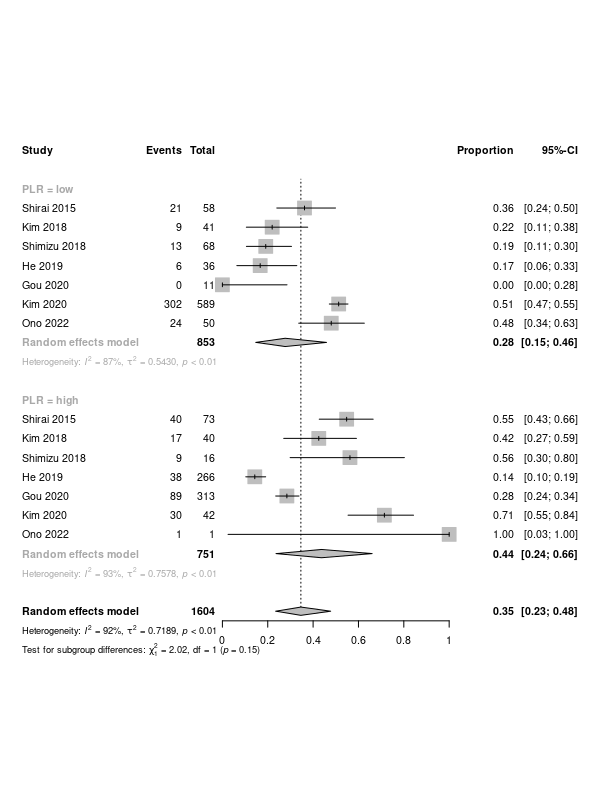


**Supplementary Figure 5.** Subgroup analysis of pooled rate of early recurrence based on Neutrophil to Lymphocyte Ratio.
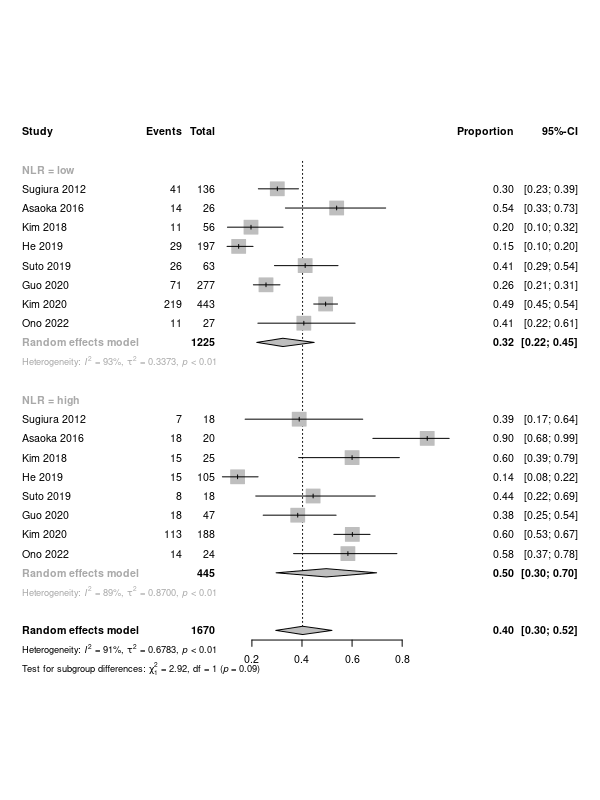


Supplementary Figure 6. Subgroup analysis of pooled rate of early recurrence based on Systemic-Immune-Inflammation Index
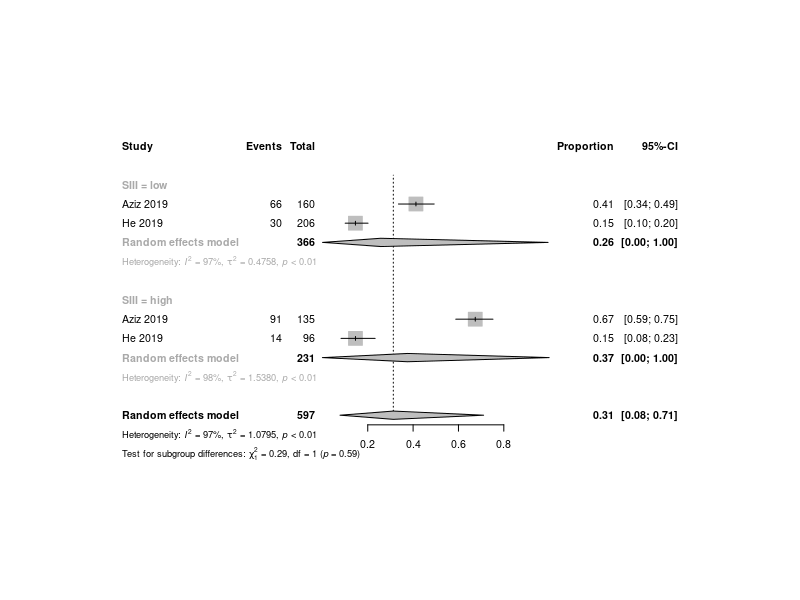


Supplementary Figure 7. Predictive role of Modified Glasgow Prognostic Score in early recurrence after pancreatectomy based on preoperative treatment subgroup analysis


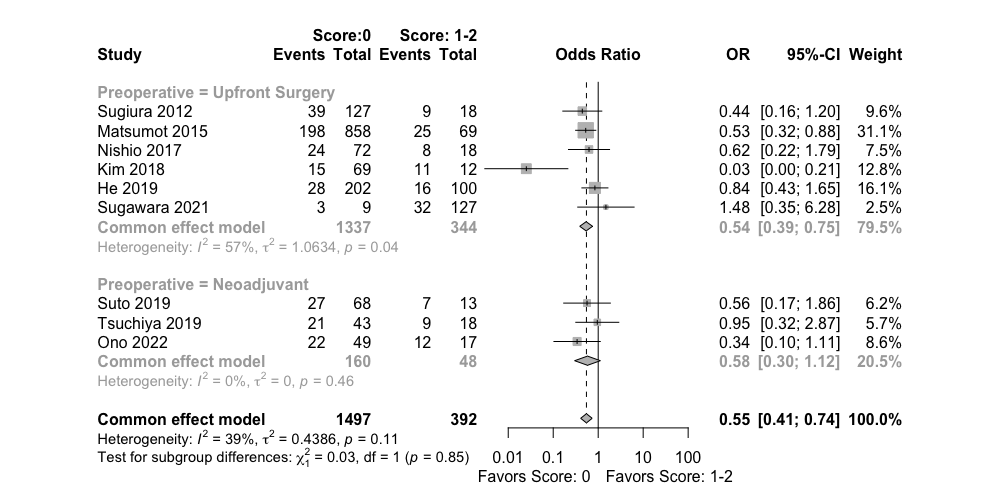


Supplementary Figure 8. Predictive role of Prognostic Nutritional Index in early recurrence after pancreatectomy based on preoperative treatment subgroup analysis


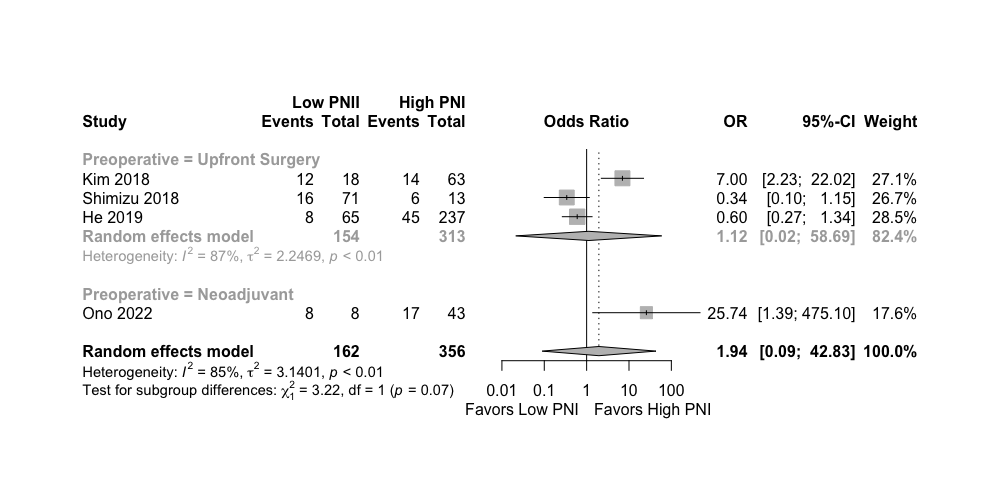


Supplementary Figure 9. Predictive role of Platelet to Lymphocyte Ratio in early recurrence after pancreatectomy based on preoperative treatment subgroup analysis.


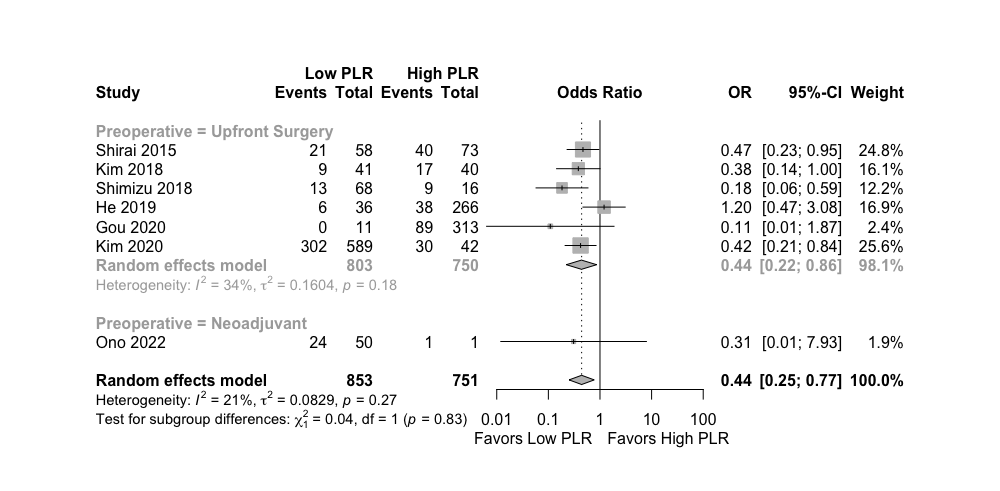


Supplementary Figure 10. Predictive role of Neutrophil to Lymphocyte Ratio in early recurrence after pancreatectomy based on preoperative treatment subgroup analysis.


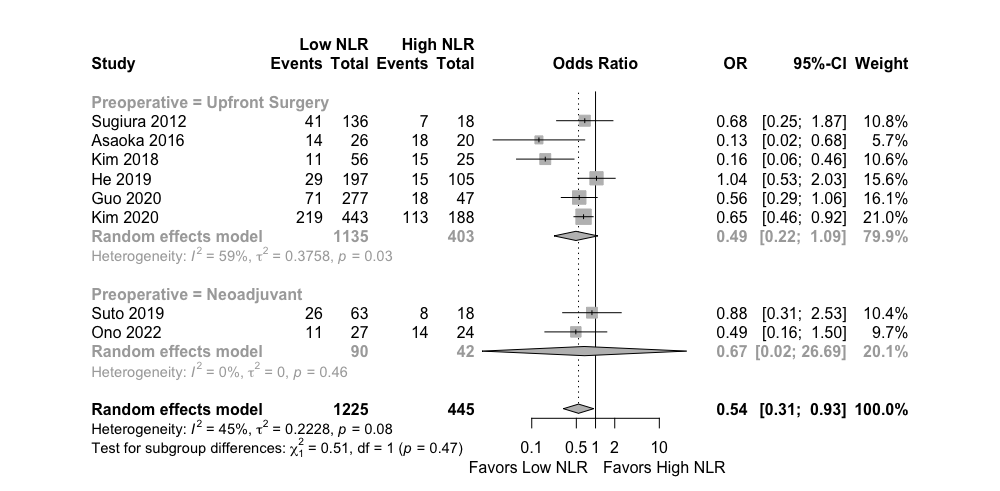

Supplement: Supplementary file 1 — Data S1. [file CAM4-13-e70352-s001.docx]
